# Prognostic Comparison Between Liver Resection and Transcatheter Arterial Chemoembolization for Hepatocellular Carcinoma Patients With Bile Duct Tumor Thrombus: A Propensity-Score Matching Analysis

**DOI:** 10.3389/fonc.2022.835559

**Published:** 2022-03-15

**Authors:** Zong-Han Liu, Ju-Xian Sun, Jin-Kai Feng, Shi-Ye Yang, Zhen-Hua Chen, Chang Liu, Zong-Tao Chai, Fei-Fei Mao, Wei-Xing Guo, Jie Shi, Shu-Qun Cheng

**Affiliations:** ^1^ Department of Hepatic Surgery VI, Eastern Hepatobiliary Surgery Hospital, Second Military Medical University, Shanghai, China; ^2^ Department of General Surgery, Zhejiang Provincial Armed Police Corps Hospital, Hangzhou, China; ^3^ Tongji University Cancer Center, Shanghai Tenth People’s Hospital, Tongji University, Shanghai, China

**Keywords:** hepatocellular carcinoma (HCC), bile duct tumor thrombus (BDTT), liver resection, transcatheter arterial chemoembolization (TACE), prognosis

## Abstract

**Background:**

Hepatocellular carcinoma (HCC) with bile duct tumor thrombus (BDTT) is rare. The aim of this study is to evaluate the long-term prognosis of liver resection (LR) versus transcatheter arterial chemoembolization (TACE) in these patients.

**Methods:**

Data from HCC patients with BDTT who underwent liver resection and TACE were analyzed respectively. Propensity score matching (PSM) analysis was performed in these patients.

**Results:**

A total of 145 HCC patients with BDTT were divided into two groups: the LR group (n = 105) and the TACE group (n = 40). The median OS in the LR group was 8.0 months longer than that in the TACE group before PSM (21.0 vs. 13.0 months, P <0.001) and 9.0 months longer after PSM (20.0 vs. 11.0 months, P <0.001). The median DFS in the LR group was 3.5 months longer than that in the TACE group before PSM (7.0 vs. 3.5 months, P = 0.007) and 5 months longer after PSM (7.0 vs. 2.0 months, P = 0.007).

**Conclusion:**

If surgery is technically feasible, liver resection provides better prognosis for HCC patients with BDTT compared with TACE.

## Introduction

Bile duct tumor thrombus (BDTT) involves invasion of hepatocellular carcinoma (HCC) into the biliary tree (1), and it is relatively uncommon with a reported incidence from 1.2 to 12.9% (2–5). The median survival of HCC patients with BDTT treated with conservative management is 1.6–4.3 months (6). However, most current practice guidelines do not provide any recommendations for this particular subgroup (7), except the Liver Cancer Study Group of Japan (LCSGJ) which considers BDTT as a poor prognostic sign and incorporates BDTT into the HCC staging system (8). Therefore, there is still a controversy over the treatment and prognosis of HCC with BDTT.

Most BDTT patients are hospitalized for obstructive jaundice, which in such circumstances more aggressive treatments should be considered, but TACE is considered as one of the feasible treatments and has rendered a favorable long-term survival outcome compared with the best conservative management (9, 10). With a better understanding of BDTT and the progress in diagnosis and surgical techniques, an increasing number of groups evaluated the prognosis of HCC patients with BDTT who underwent liver resection and reported the 3-year survival rates ranging from 24.3 to 77% (11–14), which were higher than those of the conservative therapy. However, clinical studies regarding the prognostic difference between liver resection (LR) and TACE for BDTT are limited, while extensive studies have been done in HCC with portal vein tumor thrombosis (PVTT) or hepatic vein tumor thrombus (HVTT) (15, 16). Thus, a study on the efficacy difference between surgery and TACE for HCC patients associated with BDTT is important.

In this study, we retrospectively analyzed the clinical and pathological features of HCC patients with BDTT who underwent liver resection or TACE, with the purpose of exploring the potential benefits of liver resection compared with TACE and of identifying pre-treatment factors which can impact the clinical decision-makings.

## Materials and Methods

### Ethical Statement

This study was in accordance with the Declaration of Helsinki (as revised in 2013) and was approved by the Institutional Review Board of the Eastern Hepatobiliary Surgery Hospital. Written informed consent was obtained from all patients before the treatment.

### Patients

This retrospective study included patients who were diagnosed with HCC with BDTT between November 2009 and August 2018. The diagnosis of HCC was confirmed by two coincidental imaging techniques (ultrasonography [US], contrast-enhanced computed tomography [CT], and/or magnetic resonance imaging [MRI]), or one typical radiographic imaging characteristic of HCC in conjunction with an abnormal serum α-fetoprotein (AFP) level of higher than 400 ng/ml. The presence of BDTT was determined by the clinical manifestations like obstructive jaundice in association with typical imaging findings (e.g., biliary occupation, bile duct dilatation). If necessary, endoscopic retrograde cholangiopancreatography (ERCP) or magnetic resonance cholangiopancreatography (MRCP) was used to make a definite diagnosis and evaluate the extent of BDTT.

### Eligibility Criteria

The inclusion criteria were patients who (I) were diagnosed with HCC with BDTT using the diagnostic criteria as mentioned above; (II) had liver function of Child–Pugh class A or B; (III) Eastern Cooperative Oncology Group (ECOG) performance status of 0–2; and (IV) did not accept previous anti-cancer treatment. The exclusion criteria included: (I) liver function of Child–Pugh class C at the time of the first diagnosis; (II) underwent prior or concomitant other anti-tumor treatment (e.g., local ablation, percutaneous ethanol injection, systemic chemotherapy); (III) refractory ascites, hepatic encephalopathy or coagulopathy; (IV) esophagogastric variceal hemorrhage; (V) presence of distant metastasis; (VI) combined with other serious respiratory or cardiovascular comorbidities; and (VII) incomplete clinical data or lost to follow-up. Finally, a total of 145 HCC patients with BDTT who underwent LR or TACE were enrolled in this study.

### Surgical Procedure

The patients in the LR group all received open surgery. The surgical procedures comprised of the liver resection of tumors and the removal of BDTT. The operative methods of liver resection have been described in our previous studies (17, 18). For the management of BDTT, two surgical procedures were adopted based on the relationship of BDTT with the bile duct wall. A unique technique, similar to the bile duct preserving surgery reported by Yamamoto et al. (19), was adopted to peel off the tumor thrombus if the BDTT was adhered loosely to the bile duct wall. If the BDTT was adhered to the bile duct wall tightly, extrahepatic bile duct was resected and bilioenteric anastomosis and reconstruction was fashioned with Roux-en-Y hepaticojejunostomy (20). After the removal of BDTT, the ductal lumen was carefully inspected under intraoperative cholangiography to verify that no residual tumor thrombus was present. The specimens of HCC and invaded bile duct were labeled and delivered for histopathological examination.

### TACE Procedure

TACE was performed in patients who were not eligible or unwilling to receive liver resection. TACE was carried out with the techniques described previously (21, 22). Angiography of the superior mesenteric and hepatic artery was performed to assess the vascular anatomy, portal vein patency, and tumor vascularity. The contrast medium was injected *via* a selective 5-F RH catheter (Cook, Bloomington, IN) through the sectoral, segmental, or subsegmental hepatic arteries based on the size, location, arterial supply of the tumor, and hepatic functional reserve. An emulsion of 20 to 60 mg doxorubicin hydrochloride, cisplatin (5 mg), and 5 to 30 ml lipiodol (Lipiodol; Ultra-Fluide, Guerbet, Aulnay-Sous-Bois, France) was injected through the catheter. Gelfoam fragments were then injected to embolize the tumor-feeding arteries. The dosages of lipiodol and doxorubicin were determined by tumor size, vascularity, presence of an arterioportal shunt, and underlying liver function.

### Follow-Up

Postoperative surveillance and management protocol of patients were uniformly formulated. Generally, patients were regularly followed up at the outpatient clinic once every one to two months after discharge until death or dropout from the follow-up program. The routine follow-up items included laboratory tests (complete blood count, biochemical index, AFP, hepatitis viral screening) and abdominal US. If recurrence was highly suspected, contrast-enhanced CT or MRI was necessary to be undertaken. When recurrence was clinically ascertained, repeated surgical resection or non-surgical treatments like TACE and sorafenib were actively administered according to the general status, residual liver function and recurrence pattern of patients. This study was censored on May 31, 2020.

### Statistical Analysis

Statistical analyses of categorical or continuous variables were conducted using the χ^2^ or Fisher exact test. Survival estimates were calculated by the Kaplan–Meier method and compared by a log-rank test. Two-tailed P <0.05 was considered statistically significant in all analyses. The Cox proportional hazard model was performed to identify independent prognostic factors of OS and DFS. Subgroup analyses were assessed by the Kaplan–Meier methods, and the evaluation of each median with hazard ratio and 95% confidence interval (CI) between the LR and TACE groups were displayed on forest plots. PSM (Propensity Score Matching) was performed to decrease the confounding effects and balance the baseline of the two groups. A 1:2 match between the LR and TACE groups was done using the nearest neighbor method with a caliber of 0.2. Statistical comparisons of variables were performed with the SPSS software (Version 24.0, IBM, Armonk, New York, USA). PSM was conducted *via* MatchIt package of the R program, Version 3.4.3 (R Development Team, Vienna, Austria).

## Results

### Patient Characteristics

**Figure 1:**
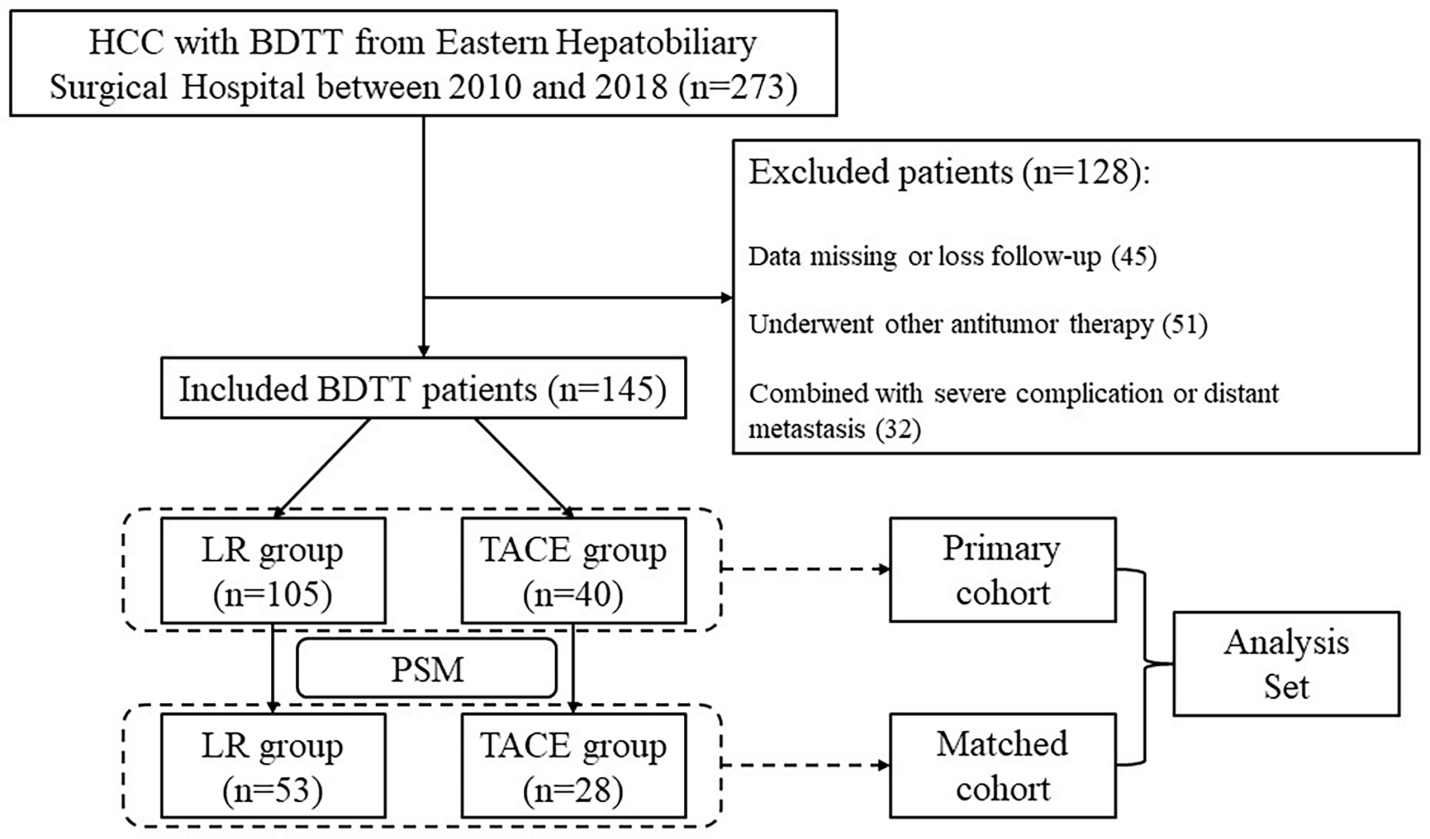
Flowchart to select eligible HCC patients with BDTT for the study. HCC, hepatocellular carcinoma; BDTT, bile duct tumor thrombus; LR, liver resection; TACE, transcatheter arterial chemoembolization; RFA, radiofrequency ablation; RT, radiation therapy; PSM, propensity score matching.

Of 273 patients, 145 HCC patients with BDTT were eligible to be included in this study. Of these, 105 received LR and 40 received TACE before PSM (**Figure 1**). The baseline characteristics of HCC patients with BDTT before PSM in the LR and TACE groups are shown in **Table 1**. Compared with the TACE group, the LR group had a lower rate of HBV infection (60% vs 77.5%, *P* = 0.049), a less frequency of HBeAg positivity (6.7% vs 22.5%, *P* = 0.014), a higher level of albumin (ALB) (39.6 vs 37.1 g/L, *P* = 0.040), lower prothrombin time (PT) (11.7 vs 12.3 s, *P* = 0.002), a lower rate of multiple tumors (18.1% vs 35%, *P* = 0.030), and a higher probability of absence of macrovascular invasion (96.2% vs 75%, *P <*0.001). After PSM, there were 53 patients in the LR group and 28 patients in the TACE group, respectively, and all these clinicopathological characteristics were balanced between the two groups (all P >0.05, **Table 1**).

**Table 1:** Baseline characteristics of HCC patients with BDTT before and after PSM.

Clinical variables	Before PSM			After PSM		
	LR group (n = 105)	TACE group (n = 40)	*P*	LR group (n = 53)	TACE group (n = 28)	*P*
Age, years	54 (47–62)	55 (45–62)	0.827	50 (47–60)	55 (45–62)	0.676
Sex			0.548			0.758
Male	86 (81.9%)	31 (77.5%)		45 (84.9%)	23 (82.1%)	
Female	19 (18.1%)	9 (22.5%)		8 (15.1%)	5 (17.9%)	
Child–Pugh class			0.266			0.252
A	63 (60.0%)	28 (70.0%)		29 (54.7%)	19 (67.9%)	
B	42 (40.0%)	12 (30.0%)		24 (45.3%)	9 (32.1%)	
HBsAg			**0.049**			0.622
Positive	63 (60.0%)	31 (77.5%)		37 (69.8%)	21 (75.0%)	
Negative	42 (40.0%)	9 (22.5%)		16 (30.2%)	7 (25.0%)	
HBeAg			**0.014**			1.000
Positive	7 (6.7%)	9 (22.5%)		7 (13.2%)	3 (10.7%)	
Negative	98 (93.3%)	31 (77.5%)		46 (86.8%)	25 (89.3%)	
Anti-HCV			0.305			1.000
Positive	2 (1.9%)	2 (5.0%)		1 (1.9%)	1 (3.6%)	
Negative	103 (98.1%)	38 (95.0%)		52 (98.1%)	27 (96.4%)	
HBV DNA, copies/ml			0.155			0.836
≤1,000	85 (81.0%)	28 (70.0%)		39 (73.6%)	20 (71.4%)	
>1,000	20 (19.0%)	12 (30.0%)		14 (26.4%)	8 (28.6%)	
WBC, 10^9^/L	5.4 (4.4–7.4)	5.2 (3.8–7.2)	0.320	5.9 (4.5–7.4)	5.2 (3.8–7.8)	0.421
HGB, g/L	129 (16)	125 (18)	0.165	129 (18)	125 (18)	0.368
PLT, 10^9^/L	180 (142–276)	169 (120–220)	0.125	173 (140–302)	185 (121–256)	0.545
ALB, g/L	39.6 (37.0–42.2)	37.1 (34.6–41.0)	**0.040**	39.0 (36.4–42.3)	37.2 (34.5–40.4)	0.113
TBIL, umol/L	21.4 (13.3–117.5)	27.3 (19.7–44.1)	0.676	31.5 (14.4–161.3)	28.6 (21.8-44.1)	0.659
ALT, U/L	62.8 (31.5–104.0)	50.5 (29.8–104.8)	0.580	64.0 (41.5–105.5)	50.5 (33.3–101.2)	0.379
GGT, U/L	313.0 (193.0–587.0)	324.0 (188.3–523.3)	0.963	307.0 (205.5–562.5)	343.0 (210.8–554.0)	0.743
ALP, U/L	189.0 (116.5–307.5)	179.5 (133.5–269.5)	0.907	189.0 (126.0–321.5)	169.0 (125.8–269.5)	0.487
PT, s	11.7 (11.2–12.4)	12.3 (11.5–13.8)	**0.002**	12.2 (11.5–13.0)	12.3 (11.3–13.5)	0.350
Scr, umol/L	65.5 (12.2)	65.3 (14.1)	0.927	65.8 (13.1)	68.4 (15.0)	0.415
CA 19-9, U/ml	56.3 (19.6–190.2)	71.5 (29.9–145.2)	0.540	78.5 (26.6–177.8)	71.5 (27.7–138.9)	0.800
AFP, ng/ml			0.186			0.114
≤400	75 (71.4%)	24 (60.0%)		36 (67.9%)	14 (50.0%)	
>400	30 (28.6%)	16 (40.0%)		17 (32.1%)	14 (50.0%)	
Tumor diameter, cm			0.071			0.976
≤5	57 (54.3%)	15 (37.5%)		21 (39.6%)	11 (39.3%)	
>5	48 (45.7%)	25 (62.5%)		32 (60.4%)	17 (60.7%)	
Tumor number			**0.030**			0.612
Solitary	86 (81.9%)	26 (65.0%)		37 (69.8%)	18 (64.3%)	
Multiple	19 (18.1%)	14 (35.0%)		16 (30.2%)	10 (35.7%)	
Macrovascular invasion			**<0.001**			0.688
Presence	4 (3.8%)	10 (25.0%)		4 (7.5%)	3 (10.7%)	
Absence	101 (96.2%)	30 (75.0%)		49 (92.5%)	25 (89.3%)	

HCC, hepatocellular carcinoma; BDTT, bile duct tumor thrombus; PA-TACE, postoperative adjuvant transarterial chemoembolization; LR, liver resection; HBsAg, hepatitis-B antigen; ALB, albumin; ALT, alanine aminotransferase; TBil, total bilirubin; PT, prothrombin time; AFP, alpha-fetoprotein; EBDR, extrahepatic bile duct resection; PVTT, portal vein tumor thrombus.

Statistically significant values are depicted as bold format.

### Survival Analysis Before and After PSM

**Figure 2:**
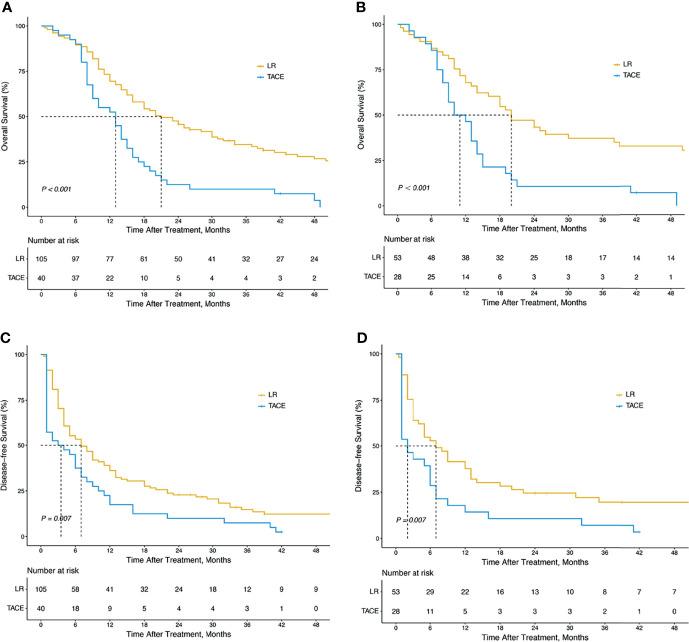
Kaplan–Meier survival curves of OS and DFS in all HCC patients with BDTT. OS for HCC patients with BDTT in the LR and TACE groups (105 patients vs. 40 patients) before PSM **(A)** (P < 0.001); DFS in the LR and TACE groups (105 patients vs. 40 patients) before PSM **(C)** (P < 0.001); OS for HCC patients with BDTT in the LR and TACE groups (53 patients vs. 28 patients) after PSM **(B)** (P = 0.007); DFS in the LR and TACE groups (53 patients vs. 28 patients) after PSM **(D)** (P = 0.007). OS, overall survival; DFS, disease-free survival; HCC, hepatocellular carcinoma; BDTT, bile duct tumor thrombus; LR, liver resection; TACE, transcatheter arterial chemoembolization.

As shown in **Figure 2**, the overall survival (OS) of patients who underwent LR was significantly longer than that of patients who underwent TACE (median OS time, 21.0 months vs. 13.0 months; 1-year, 69.5% vs.52.5%; 2-year, 45.7% vs.12.5%; 3-year, 34.6% vs.10.0%; *P <*0.001; **Figure 2A**). Similarly, the disease-free survival (DFS) of the LR group was substantially longer than that of the TACE group (median DFS time, 7.0 months vs. 3.5 months; 1-year, 38.3% vs. 17.5%; 2-year, 23.2% vs. 10.0%; 3-year, 12.1% vs.7.5%; *P* = 0.007; **Figure 2C**).

After 1:2 PSM, the long-term prognosis of the LR group was also significantly better than the TACE group (for OS: median OS time, 20.0 months vs. 11.0 months; 1-year, 67.9% vs. 46,4%; 2-year, 43.4% vs. 14.3%; 3-year 37.3% vs. 10.7%; *P <*0.001; **Figure 2B**; for DFS: median DFS time, 7.0 months vs. 2.0 months; 1-year, 38.5% vs. 14.3%; 2-year, 25.0% vs. 14.3%; 3-year, 20.0% vs. 7.1%; *P* = 0.007; **Figure 2D**).

### Risk Factors Associated With OS and DFS for All the Patients

Before PSM, treatment allocation (HR = 0.429, 95% CI = 0.288–0.637), Child–Pugh class (HR = 1.588, 95% CI = 1.101–2.292), HBsAg (HR = 1.411, 95% CI = 0.965–2.063), HGB level (HR = 0.985, 95% CI = 0.972–0.997), ALB level (HR = 0.958, 95% CI = 0.935–0.981), TBIL level (HR = 1.002, 95% CI = 1.000–1.003), tumor number (HR = 2.066, 95% CI = 1.354–3.154) and major vascular invasion (HR = 1.797, 95% CI = 1.021–3.161) were identified as potential risk factors of OS (**Table 2**).Whereas treatment allocation (HR = 0.520, 95% CI = 0.331–0.815), HGB level (HR = 0.985, 95% CI = 0.973–0.999), ALB level (HR = 0.967, 95% CI = 0.936–1.000) and tumor number (HR = 1.982, 95% CI = 1.247–3.152) were independent risk factors of OS (**Table 2**).

**Table 2:** Prognostic factors for overall survival and Progress-free survival before PSM.

Clinical variables	Overall survival				Progress-free survival			
	Univariate		Multivariate		Univariate		Multivariate	
	HR (95% CI)	*P*	HR (95% CI)	*P*	HR (95% CI)	*P*	HR (95% CI)	*P*
Treatment allocation, LR vs. TACE	0.429 (0.288–0.637)	**<0.001**	0.520 (0.331–0.815)	**0.004**	0.614 (0.421–0.897)	**0.012**	0.618 (0.420–0.909)	**0.015**
Age (per 1 year increase)	0.993 (0.975–1.012)	0.490			0.990 (0.973–1.007)	0.256		
Sex, male vs. female	1.204 (0.766–1.892)	0.421			1.099 (0.712–1.694)	0.670		
Child–Pugh class, B vs. A	1.588 (1.101–2.292)	**0.013**			1.357 (0.950–1.939)	**0.093**		
HBsAg, positive vs. negative	1.411 (0.965–2.063)	**0.076**			1.385 (0.960–1.996)	**0.081**		
HBeAg, positive vs. negative	1.122 (0.630–1.998)	0.696			1.287 (0.723–2.290)	0.392		
Anti-HCV, positive vs. negative	1.030 (0.379–2.793)	0.954			1.907 (0.606–6.002)	0.270		
HBV DNA, >1,000 vs. ≤1,000 copies/ml	1.104 (0.722–1.689)	0.648			1.009 (0.663–1.537)	0.966		
WBC (per 1 ∗ 10^9^/L increase)	1.047 (0.964–1.137)	0.273			1.037 (0.957–1.123)	0.347		
HGB (per 1 g/L increase)	0.985 (0.972–0.997)	**0.015**	0.985 (0.973–0.999)	**0.029**	0.992 (0.980–1.004)	0.186		
PLT (per 1 ∗ 10^9^/L increase)	0.999 (0.997–1.002)	0.635			1.000 (0.998–1.002)	0.827		
ALB (per 1 g/L increase)	0.958 (0.935–0.981)	**<0.001**	0.967 (0.936–1.000)	**0.049**	0.973 (0.949–0.997)	**0.028**		
TBIL (per 1 umol/L increase)	1.002 (1.000–1.003)	**0.055**			1.002 (1.000–1.003)	**0.027**	1.003 (1.001–1.004)	**0.004**
ALT (per 1 U/L increase)	0.998 (0.995–1.001)	0.118			0.999 (0.996–1.001)	0.237		
GGT (per 1 U/L increase)	1.000 (0.999–1.000)	0.912			1.000 (1.000–1.000)	0.924		
ALP (per 1 U/L increase)	1.000 (0.999–1.002)	0.495			1.000 (0.999–1.001)	0.552		
PT (per 1 s increase)	1.090 (0.978–1.215)	0.120			1.059 (0.953–1.177)	0.283		
Scr (per 1 umol/L increase)	0.990 (0.975–1.004)	0.165			0.995 (0.981–1.009)	0.491		
CA 19-9 (per 1 U/ml increase)	1.001 (1.000–1.001)	0.105			1.000 (1.000–1.001)	0.172		
AFP, >400 vs. ≤400 ng/ml	1.168 (0.788–1.730)	0.439			1.147 (0.788-1.668)	0.475		
Tumor diameter, >5 vs. ≤5 cm	1.069 (0.746–1.532)	0.715			1.039 (0.735–1.468)	0.829		
Tumor number, multiple vs. solitary	2.066 (1.354–3.154)	**0.001**	1.982 (1.247–3.152)	**0.004**	1.545 (1.022–2.336)	**0.039**	1.625 (1.053–2.507)	**0.028**
Major vascular invasion, yes vs. no	1.797 (1.021–3.161)	**0.042**			1.429 (0.819–2.493)	0.209		

HCC, hepatocellular carcinoma; BDTT, bile duct tumor thrombus; TACE, transarterial chemoembolization; CM, conservative management; HBsAg, hepatitis B surface antigen; HBeAg, hepatitis B e antigen; HCV, hepatitis C virus; HBV DNA; hepatitis B virus deoxyribonucleic acid; WBC, white blood cell; HGB, hemoglobin; PLT, platelet; ALB, albumin; TBIL, total bilirubin; ALT, alanine aminotransferase; GGT, γ-glutamyltransferase; ALP, alkaline phosphatase; PT, prothrombin time; Scr, serum creatinine; CA19-9, carbohydrate antigen 19-9; AFP, α-fetoprotein.

Statistically significant values are depicted as bold format.

Treatment allocation (HR = 0.614, 95% CI = 0.421–0.897), Child–Pugh class (HR = 1.357, 95% CI = 0.960–1.939), HBsAg (HR = 1.385, 95% CI = 0.960–1.996), ALB level (HR = 0.973, 95% CI = 0.949–0.997), TBIL level (HR = 1.002, 95% CI = 1.000–1.003) and tumor number (HR = 1.545, 95% CI=1.022–2.336) were potential risk factors of DFS (**Table 2**). Whereas treatment allocation (HR = 0.614, 95% CI = 0.421–0.897), TBIL level (HR = 1.003, 95% CI = 1.001–1.004) and tumor number (HR = 1.625, 95% CI = 1.053–2.507) were independent risk factors of DFS (**Table 2**).

After PSM, treatment allocation (HR = 0.406, 95% CI = 0.242–0.680), Child–Pugh class (HR = 1.740, 95% CI = 1.061–2.854), HGB level (HR = 0.984, 95% CI = 0.968–0.999), ALB level (HR = 0.937, 95% CI = 0.887–0.990), tumor number (HR = 1.776, 95% CI = 1.057–2.983) and major vascular invasion (HR = 1.992, 95% CI = 0.894–4.438) were potential risk factors of OS (**Table 3**). Whereas treatment allocation (HR = 0.429, 95% CI = 0.241–0.762), Child–Pugh class (HR = 2.131, 95% CI = 1.179–3.852), HGB level (HR = 0.982, 95% CI = 0.966–0.999) and tumor number (HR = 2.154, 95% CI = 1.184–3.919) were independent risk factors of OS (**Table 3**).

**Table 3:** Prognostic factors for overall survival and Progress-free survival after PSM.

Clinical variables	Overall survival				Progress-free survival			
	Univariate		Multivariate		Univariate		Multivariate	
	HR (95% CI)	*P*	HR (95% CI)	*P*	HR (95% CI)	*P*	HR (95% CI)	*P*
Treatment allocation, LR vs. TACE	0.406 (0.242–0.680)	**0.001**	0.429 (0.241–0.762)	**0.004**	0.538 (0.330–0.878)	**0.013**	0.479 (0.285–0.804)	**0.005**
Age (per 1 year increase)	0.993 (0.968–1.017)	0.554			0.998 (0.966–1.010)	0.275		
Sex, male vs. female	1.002 (0.509–1.971)	0.996			1.043 (0.545–1.995)	0.900		
Child–Pugh class, B vs. A	1.740 (1.061–2.854)	**0.028**	2.131 (1.179–3.852)	**0.012**	1.544 (0.952–2.502)	**0.078**		
HBsAg, positive vs. negative	1.472 (0.835–2.597)	0.181			1.577 (0.909–2.734)	0.105		
HBeAg, positive vs. negative	1.138 (0.518–2.499)	0.747			1.594 (0.727–3.495)	0.245		
Anti-HCV, positive vs. negative	1.099 (0.268–4.501)	0.896			1.601 (0.391–6.564)	0.513		
HBV DNA, >1,000 vs. ≤1,000 copies/ml	1.129 (0.654–1.949)	0.662			1.106 (0.651–1.878)	0.710		
WBC (per 1 ∗ 10^9^/L increase)	1.055 (0.951–1.169)	0.313			1.061 (0.960–1.173)	0.248		
HGB (per 1 g/L increase)	0.984 (0.968–0.999)	**0.040**	0.982 (0.966–0.999)	**0.033**	0.988 (0.974–1.003)	0.123		
PLT (per 1 ∗ 10^9^/L increase)	1.000 (0.997–1.002)	0.806			1.000 (0.997–1.002)	0.937		
ALB (per 1 g/L increase)	0.937 (0.887–0.990)	**0.020**			0.960 (0.913–1.009)	0.108		
TBIL (per 1 umol/L increase)	1.001 (0.999–1.003)	0.241			1.002 (1.000–1.004)	**0.063**		
ALT (per 1 U/L increase)	0.998 (0.994–1.001)	0.246			0.998 (0.995–1.001)	0.269		
GGT (per 1 U/L increase)	1.000 (0.999–1.001)	0.716			1.000 (0.999–1.001)	0.903		
ALP (per 1 U/L increase)	1.000 (0.998–1.002)	0.653			1.000 (0.998–1.002)	0.728		
PT (per 1 s increase)	1.169 (0.953–1.434)	0.133			1.171 (0.959–1.429)	0.121		
Scr (per 1 umol/L increase)	0.997 (0.978–1.015)	0.729			0.996 (0.978–1.014)	0.646		
CA 19-9 (per 1 U/ml increase)	1.000 (0.999–1.001)	0.708			1.000 (0.999–1.001)	0.515		
AFP, >400 vs. ≤400 ng/ml	1.212 (0.732–2.006)	0.455			1.052 (0.644–1.717)	0.839		
Tumor diameter, >5 vs. ≤5 cm	1.084 (0.660–1.780)	0.751			1.009 (0.624–1.632)	0.971		
Tumor number, multiple vs. solitary	1.776 (1.057–2.983)	**0.030**	2.154 (1.184–3.919)	**0.012**	1.489 (0.897–2.473)	0.124		
Major vascular invasion, yes vs. no	1.992 (0.894–4.438)	**0.092**			2.072 (0.929–4.619)	**0.075**		

HCC, hepatocellular carcinoma; BDTT, bile duct tumor thrombus; TACE, transarterial chemoembolization; CM, conservative management; HBsAg, hepatitis B surface antigen; HBeAg, hepatitis B e antigen; HCV, hepatitis C virus; HBV DNA; hepatitis B virus deoxyribonucleic acid; WBC, white blood cell; HGB, hemoglobin; PLT, platelet; ALB, albumin; TBIL, total bilirubin; ALT, alanine aminotransferase; GGT, γ-glutamyltransferase; ALP, alkaline phosphatase; PT, prothrombin time; Scr, serum creatinine; CA19-9, carbohydrate antigen 19-9; AFP, α-fetoprotein. Statistically significant values are depicted as bold format.

Treatment allocation (HR = 0.538, 95% CI = 0.330–0.878), Child–Pugh class (HR = 1.544, 95% CI = 0.952–2.502), TBIL level (HR = 1.002, 95% CI = 1.000–1.004) and major vascular invasion (HR = 2.072, 95% CI = 0.929–4.619) were potential risk factors of DFS (**Table 3**). Whereas treatment allocation (HR = 0.479, 95% CI = 0.285–0.804) was an independent risk factor of DFS (**Table 3**).

### Subgroup Analysis

As shown in **Supplementary Figure S1**, the patients before PSM derived significant OS benefits from LR if they were Child–Pugh class A (HR = 0.27, 95% CI = 0.16_0.44), TBIL ≤34 μmol/L (HR = 0.33, 95% CI = 0.20–0.55), single tumor (HR = 0.40, 95% CI = 0.25–0.65), or no macrovascular invasion (HR = 0.40, 95% CI = 0.26–0.63). After PSM, subgroup analysis indicated that the patients had significant OS benefits from LR if they were Child–Pugh class A (HR = 0.22, 95% CI = 0.11–0.44), ALB >40 g/L (HR = 0.22, 95% CI = 0.09–0.58), single tumor (HR = 0.35, 95% CI = 0.18–0.66), or no macrovascular invasion (HR = 0.39, 95% CI = 0.23–0.68).

As shown in **Supplementary Figure S2**, the patients before PSM derived significant DFS benefits from LR if they were Child–Pugh class A (HR = 0.46, 95% CI = 0.29–0.74), ALB >40 g/L (HR = 0.50, 95% CI = 0.26–0.97), or TBIL ≤34 μmol/L (HR = 0.55, 95% CI = 0.35–0.88). After PSM, subgroup analysis indicated that the patients had significant DFS benefits from LR if they were Child–Pugh class A (HR = 0.38, 95% CI = 0.20–0.72), TBIL ≤34 μmol (HR = 0.40, 95% CI = 0.21–0.75), or no macrovascular invasion (HR = 0.53, 95% CI = 0.32–0.89).

## Discussion

Unlike other digestive system tumors which tend to invade lymph nodes, HCC is strongly prone to invade the surrounding liver vasculature (23). HCC with BDTT is one of the most rare but special type of liver cancer. Many theories had elaborated the mechanisms of BDTT; some experts believe that the occurrence of BDTT is related to the biological characteristics of the tumor, the microenvironment, and the adjacent relationship between liver cancer and bile ducts (24). Due to the complexity and rarity, most current clinical practice guidelines do not provide recommendations clearly for HCC with BDTT. Hence, there is no consensus on the optimal therapeutic protocol for BDTT.

To our knowledge, this study was the first to compare the long-term survival of HCC patients with BDTT who underwent liver resection or TACE. In this study, the baseline characteristics of the two groups were heterogeneous to some extent, which are mostly caused by the surgical tolerability of the patients. PSM method is widely used in retrospective observational studies to reduce the between-group baseline differences as much as possible and make the two groups comparable and balanced. Our study showed that the LR group had a median survival of 21.0 months and an OS rate of 34.6% at 3 years, which were similar to the results reported in other retrospective studies (4, 25–27). The TACE group had a median survival of 13.0 months and an OS rate of 10.0% at 3 years. Concordantly, after PSM, the post-treatment long-term survival of the LR group was significantly better than the TACE group. In addition, potential beneficiaries were identified using subgroup analysis stratified by risk factors related to the long-term prognosis. The results showed that patients with such clinicopathological features (single tumor, absence of macrovascular invasion, lower levels of ALB and TBIL, or Child–Pugh class A) could benefit more from liver resection over TACE. Hence, we concluded that surgical resection should be recommended to HCC patients with BDTT, especially for those with good liver function and low tumor burden.

As is known to all, most HCC patients with BDTT are hospitalized for obstructive jaundice, and this type of HCC is often called “icteric hepatoma” (28). A serum total bilirubin level higher than 51 μmol/L is always considered a relative contraindication for chemoembolization or hepatectomy (29). To be noted, the obstructive jaundice caused by BDTT is different in nature from jaundice associated with advanced liver cirrhosis or extensive tumor infiltration, which suggests the clinicopathological features of obstructive jaundice resulted from BDTT are distinct from characteristics of parenchymal cholestasis (30). There is no ideal treatment options for jaundice caused by liver dysfunction, whereas the jaundice due to BDTT could be controlled or even treatable through endoscopic or percutaneous drainage approaches (31). Consequently, the jaundice would not be an absolute surgical contraindication for BDTT, and the identification of different jaundice types is of great importance for clinical treatment decision-making. In this study, 89 (61.4%) patients were found to be associated with low TBIL level (≤34 μmol/L). Subgroup analysis showed that BDTT patients with low TBIL level could benefit more from surgical resection; therefore, effective preoperative biliary drainage to reduce TBIL level to below 34 μmol/L is essential for surgery and postoperative long-term survival. During the surgery, two operative techniques mentioned in the Surgical Procedure could decrease the recurrence and metastasis rates (32), especially the peeling off technique, because it avoids the resection of bile duct and enables the administration of postoperative adjuvant therapies against recurrence and metastasis (19).

Tumor number is one of the most important risk factors of long-term survival and is one of the origins of controversies on the management of HCC patients with BDTT. As shown in the subgroup analysis, HCC patients with BDTT could gain more survival benefits from liver resection than TACE if the tumor number is single. Tumor number reflects the tumor burden of the BDTT patients, and more tumor lesions mean insufficient normal liver volume, which poses challenges for operation and increases the risk of postoperative liver failure (33). According to the clonal origin theory of HCC, early recurrence is often associated with single center occurrence because of residual tumor seeds and early vascular invasion (34), whereas late recurrence is usually related to multiple center recurrence and liver cirrhosis. Combined with the above points, in BDTT patients with multiple tumors, the selection of treatment modalities still needs to be discussed.

This study has several limitations. First, this study is based on retrospective data which may generate selection biases. Although PSM analysis was applied to reduce potential imbalance, the two groups were not matched at a strict 1:1 ratio due to the limited BDTT cases. Second, this study was conducted in a Chinese single center with a high proportion of HBV-related HCC. The results from this study may not be suitable for HCC caused by other etiologies.

## Conclusion

In summary, when surgery is technically feasible, liver resection can provide better long-term survival outcomes for HCC patients with BDTT compared with TACE, especially for those patients whose liver function is well preserved and tumor burden is low.

## Data Availability Statement

The raw data supporting the conclusions of this article will be made available by the corresponding author, without undue reservation.

## Ethics Statement

This study was approved by the Declaration of Helsinki (as revised in 2013) and was approved by the institutional ethics board of the Eastern Hepatobiliary Surgery Hospital. The patients/participants provided their written informed consent to participate in this study.

## Author Contributions

Conception and design: S-QC, Z-HL, J-XS, J-KF, and S-YY. Administrative support: S-QC. Provision of study materials or patients: J-XS, W-XG, and JS. Collection and assembly of data: Z-TC, Z-HL, and J-KF. Data analysis and interpretation: Z-HL, J-KF, and S-YY. Statistical analysis: Z-HL, J-KF, and S-YY. All authors listed have made a substantial, direct, and intellectual contribution to the work and approved it for publication.

## Funding

The study received support from the National Natural Science Foundation of China (82172846).

## Conflict of Interest

The authors declare that the research was conducted in the absence of any commercial or financial relationships that could be construed as a potential conflict of interest.

## Publisher’s Note

All claims expressed in this article are solely those of the authors and do not necessarily represent those of their affiliated organizations, or those of the publisher, the editors and the reviewers. Any product that may be evaluated in this article, or claim that may be made by its manufacturer, is not guaranteed or endorsed by the publisher.

## Supplementary Material

Supplementary Figure S1 Subgroup analysis of MOST stratified by potential risk factors before and after PSM. MOST, median overall survival time; LR, liver resection; TACE, transcatheter arterial chemoembolization; PSM, propensity score matching; CI, confidence interval; HBsAg, hepatitis B surface antigen; HGB, hemoglobin; ALB, albumin; TBIL, total bilirubin.

Supplementary Figure S2 Subgroup analysis of MDFST stratified by potential risk factors before and after PSM. MDFST, median disease-free survival time; LR, liver resection; TACE, transcatheter arterial chemoembolization; PSM, propensity score matching; CI, confidence interval; HBsAg, hepatitis B surface antigen; ALB, albumin; TBIL, total bilirubin.
