# Supplementary figures and images for: Prognostic Comparison Between Liver Resection and Transcatheter Arterial Chemoembolization for Hepatocellular Carcinoma Patients With Bile Duct Tumor Thrombus: A Propensity-Score Matching Analysis
Source: Front Oncol. 2022 Mar 15;12:835559. doi: 10.3389/fonc.2022.835559 (PMC8964486; doi:10.3389/fonc.2022.835559)

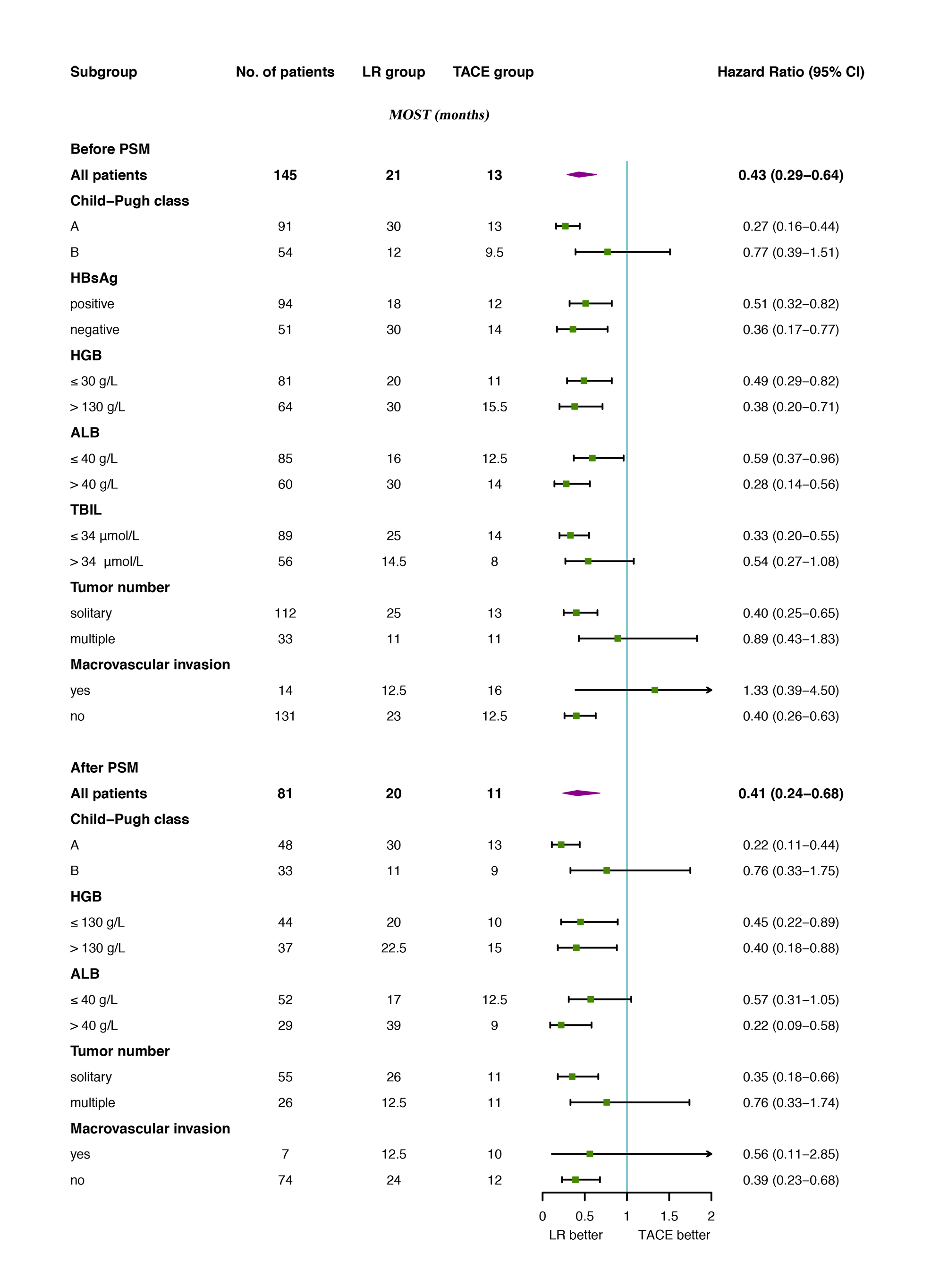

Supplement: Supplementary Figure S1 — Subgroup analysis of MOST stratified by potential risk factors before and after PSM. MOST, median overall survival time; LR, liver resection; TACE, transcatheter arterial chemoembolization; PSM, propensity score matching; CI, confidence interval; HBsAg, hepatitis B surface antigen; HGB, hemoglobin; ALB, albumin; TBIL, total bilirubin. [file Image_1.tif]

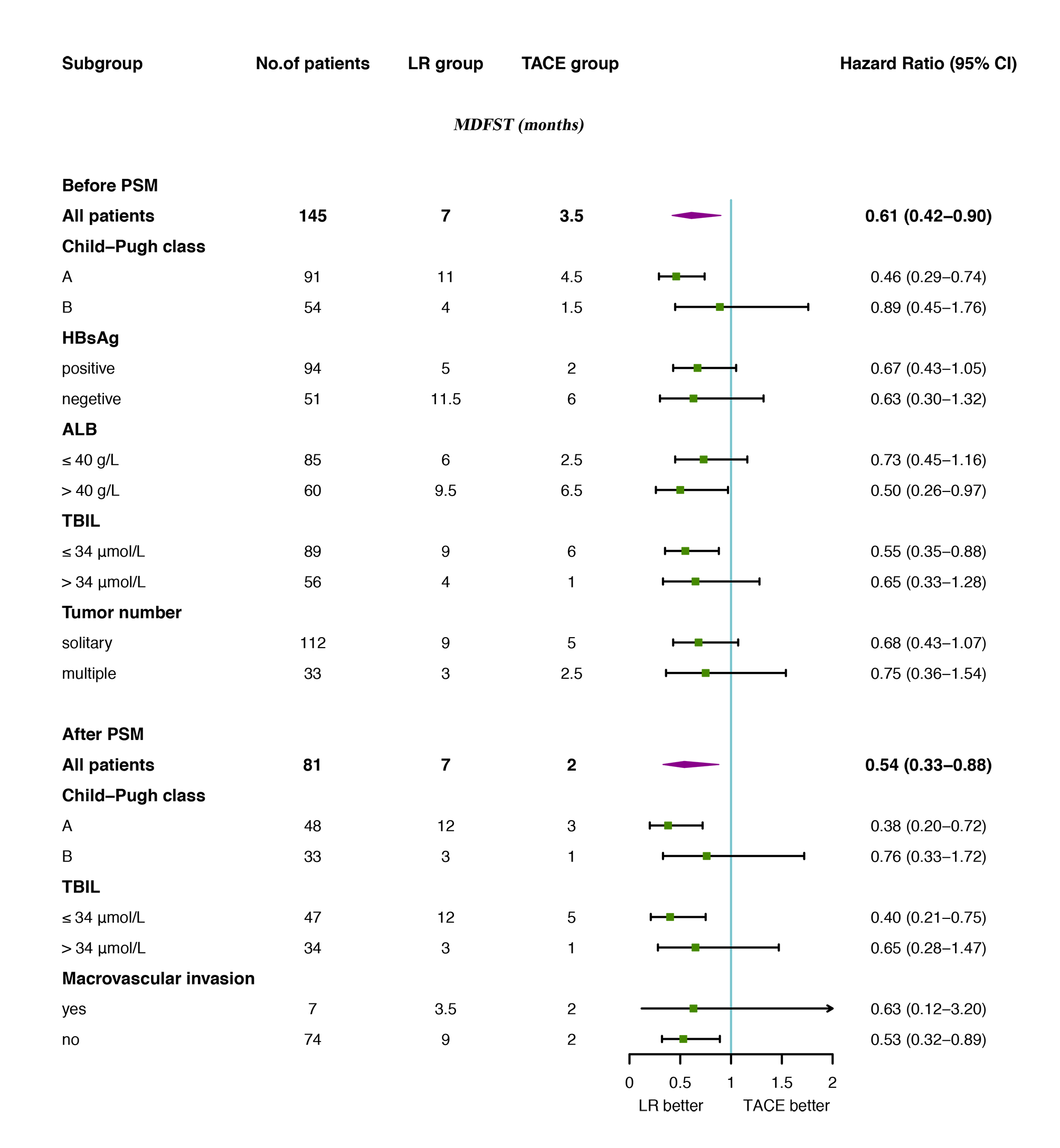

Supplement: Supplementary Figure S2 — Subgroup analysis of MDFST stratified by potential risk factors before and after PSM. MDFST, median disease-free survival time; LR, liver resection; TACE, transcatheter arterial chemoembolization; PSM, propensity score matching; CI, confidence interval; HBsAg, hepatitis B surface antigen; ALB, albumin; TBIL, total bilirubin. [file Image_2.tif]
